# Measurement of Entrance Surface Dose on an Anthropomorphic Thorax Phantom Using a Miniature Fiber-Optic Dosimeter

**DOI:** 10.3390/s140406305

**Published:** 2014-04-01

**Authors:** Wook Jae Yoo, Sang Hun Shin, Dayeong Jeon, Seunghan Hong, Hyeok In Sim, Seon Geun Kim, Kyoung Won Jang, Seunghyun Cho, Won Sik Youn, Bongsoo Lee

**Affiliations:** 1 School of Biomedical Engineering, College of Biomedical & Health Science, Research Institute of Biomedical Engineering, Konkuk University, 268 Chungwon-daero, Chungju-si, Chungcheongbuk-do, 380-701, Korea; E-Mails: wonzip@kku.ac.kr (W.J.Y.); shshin9431@gmail.com (S.H.S); jdy603@naver.com (D.J); zzang811@gmail.com (S.H.); saucony9116@msn.com (H.I.S.); chokomilkys@gmail.com (S.G.K.); kko988@kku.ac.kr (K.W.J.); 2 Department of *Organic* Materials & Fiber Engineering, College of Engineering, Soongsil University, 369 Sangdo-ro, Dongjak-gu, Seoul-si, 156-743, Korea; E-Mail: scho@ssu.ac.kr; 3 Research & Development Center, JPI Healthcare, Osongsaengmyeong 1-ro, Osong-eup, Cheongwon-gun, Chungcheongbuk-do, 363-951, Korea; E-Mail: younws@JPI.co.kr

**Keywords:** fiber-optic dosimeter, entrance surface dose, diagnostic radiology, scintillating light, anthropomorphic thorax phantom

## Abstract

A miniature fiber-optic dosimeter (FOD) system was fabricated using a plastic scintillating fiber, a plastic optical fiber, and a multi-pixel photon counter to measure real-time entrance surface dose (ESD) during radiation diagnosis. Under varying exposure parameters of a digital radiography (DR) system, we measured the scintillating light related to the ESD using the sensing probe of the FOD, which was placed at the center of the beam field on an anthropomorphic thorax phantom. Also, we obtained DR images using a flat panel detector of the DR system to evaluate the effects of the dosimeter on image artifacts during posteroanterior (PA) chest radiography. From the experimental results, the scintillation output signals of the FOD were similar to the ESDs including backscatter simultaneously obtained using a semiconductor dosimeter. We demonstrated that the proposed miniature FOD can be used to measure real-time ESDs with minimization of DR image artifacts in the X-ray energy range of diagnostic radiology.

## 1. Introduction

In diagnostic radiology and radiotherapy, measurement of the delivered dose is very important to minimize unnecessary exposure. For *in situ* dose measurement at the measuring point, many different types of dosimeters have been developed, including thermoluminescent dosimeters (TLDs), glass rod dosimeters (GRDs), radiochromic dosimetry films, metal-oxide semiconductor field-effect transistors (MOSFETs), semiconductor dosimeters (SCDs), ionization chambers (ICs), and fiber-optic dosimeters (FODs). Generally, an ideal dosimeter should have a small sized sensitive element, a tissue- or water-equivalent characteristic, and the capability of providing real-time dose monitoring for *in vivo* dosimetry. Thin dosimeters, such as TLDs, GRDs, and films, have been previously proposed for measuring dose information directly. Although these thin dosimeters can provide dose information with high spatial resolution, they cannot be used for real-time dosimetry because they require a time-consuming reading process after beam irradiation [1–3]. In the cases of a MOSFET and a SCD, their high-atomic-number materials can induce distracting image artifacts during real-time monitoring [4–6]. Normally, most ICs have a large sensitive element for in-phantom dose measurement and need a complicated correction process and frequent calibration [7,8]. Lastly, various FODs have been investigated due to their desirable dosimetric qualities, such as small volume, light weight, substantial flexibility, real-time dose monitoring at long range, and immunity to electromagnetic interference. However, while most FODs have been developed to measure real-time absorbed dose in radiotherapy [9–14], fewer studies on the use of FODs have been performed for diagnostic radiology [5,6,15–17].

In this study, a miniature FOD system that consists of a plastic scintillating fiber (PSF), a plastic optical fiber (POF), and a multi-pixel photon counter (MPPC) was developed for real-time scintillation dosimetry in diagnostic radiology. We measured the scintillating light generated from a sensing probe of the FOD during beam irradiation and compared the results with the radiation doses obtained using a conventional dosimeter.

## 2. Materials and Experimental Setup

### 2.1. Fabrication of a Sensing Probe

To produce scintillating light having dose information, as an organic scintillator, a PSF (BCF-12, Saint-Gobain Ceramic & Plastics, Hiram, OH, USA) was used. The PSF has a cylindrical shape and a core/clad structure with an outer diameter of 1 mm. The refractive indices of the core and the cladding are 1.60 and 1.49, respectively, and the numerical aperture (NA) is 0.58. The core is synthesized with polystyrene (PS) and fluorescent dopants and the cladding material is polymethylmethacrylate (PMMA). The emission peak wavelength and the decay time of the PSF are 435 nm and 2.7 ns, respectively.

A multimode POF (GH-4001, Mitsubishi Rayon, Tokyo, Japan) with a step refractive index profile is used to transmit the scintillating light from the PSF to a photon-counting device, a MPPC (S10362-11-100U, Hamamatsu Photonics, Hamamatsu, Japan) with an active area of 1 × 1 mm^2^. The diameter of the core is 0.98 mm and the outer diameter of the POF is equal to that of the PSF. The core is made of PMMA resin with a refractive index of 1.49 and the fluorinated polymer based cladding has a refractive index of 1.402; thereby the NA of this POF is 0.5. The POF is covered by a black polyethylene (PE) jacket with an outer diameter of 2.2 mm to shield light noise and to protect the POF from ambient contamination.

**Figure 1.**
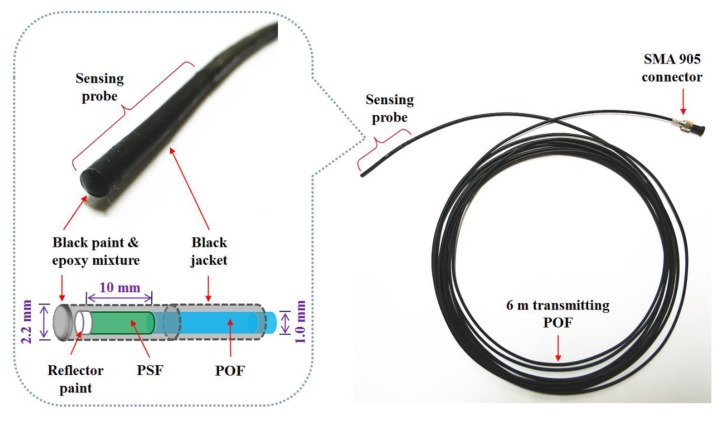
Completed sensing probe and its internal structure to produce scintillating light.

Figure 1 shows a photograph of the completed sensing probe and its internal structure. To maximize the amount of scintillating light reaching the MPPC, both ends of the PSF and POF, respectively, were polished with various types of lapping films in a regular sequence. The bared PSF with a length of 10 mm was connected to the distal end of the jacketed POF with a length of 6 m and then the uncoupled end of the PSF was coated with a titanium dioxide (TiO_2_)-based reflector paint (BC-620, Saint-Gobain Ceramic & Plastics) to increase the collection efficiency of the scintillating light. Furthermore, the outer surface of the PSF was covered with a black PE jacket and the distal end was coated by a mixture of black paint and an optical epoxy to block external light. Finally, a subminiature type A (SMA) 905 connector was installed at the opposite end of the POF to enable the sensing probe to be connected with the MPPC.

### 2.2. Experimental Setup Using a Digital Radiography System and an Anthropomorphic Thorax Phantom

**Figure 2.**
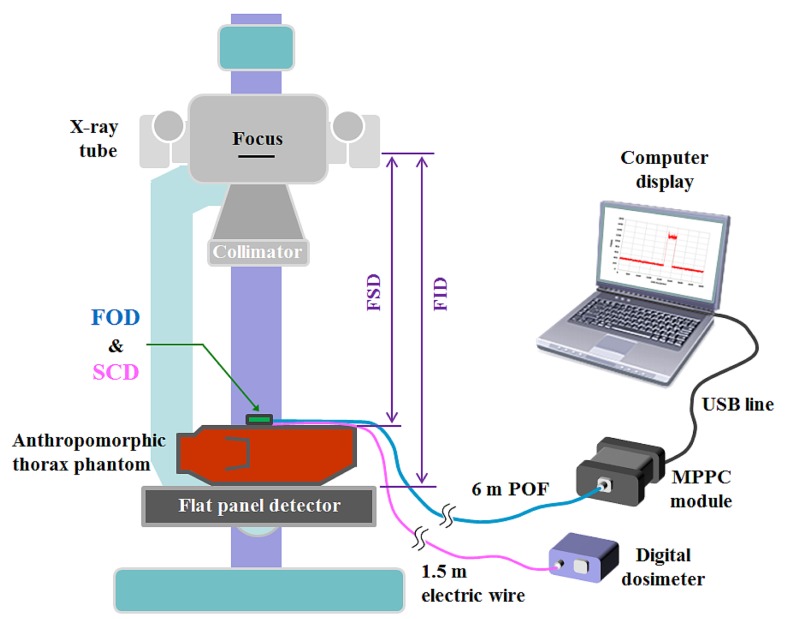
Experimental setup for measuring ESDs on the anthropomorphic thorax phantom in a DR system.

In plain radiography examinations, the patient dose is generally described as the entrance surface dose (ESD), which is measured at the center of the entrance surface in the X-ray beam field, and includes backscattered radiation from the patient [18,19]. Figure 2 shows the experimental setup for measuring ESDs in a digital radiography (DR) system using the FOD and a conventional SCD. To measure weak scintillating light transmitted from the sensing probe, we used an MPPC module (C10751-03, Hamamatsu Photonics) that has a SMA-type optical fiber adapter for fast and easy connection of an internal MPPC device (S10362-11-100U, Hamamatsu Photonics) to the sensing probe. The MPPC device with an active area of 1 × 1 mm^2^ is composed of multiple avalanche photodiode (APD) pixels and the signal output from the MPPC module is the total sum of the outputs from all APD pixels of the MPPC device.

The MPPC module has high sensitivity in a spectral response range from 320 to 900 nm and a peak sensitivity wavelength of 440 nm. At the threshold level condition of 0.5 photon equivalent (p.e.), the photon detection efficiency and the typical dark count of this MPPC module are 45% and 900 kilo counts per second (kcps), respectively. Here, p.e. indicates that one photon was detected. As a reference dosimeter, a commercially available SCD, a finger-shaped dose sensor that is connected to a digital dosimeter (Pehamed, ALPHA plus, Sulzbach, Germany) was used to compare its ESDs with the output signals of the FOD. To evaluate the performance of the fabricated FOD, an experiment was carried out using an X-ray tube (Rotanode^TM^ E7252X, Toshiba Electron Tubes & Devices, Saitama, Japan) of the DR system (Clear Vision DR 7000F, JPI Healthcare, Osong, Korea) with a focal spot of 0.6/1.2 mm, a 12° rhenium-tungsten-faced molybdenum target, permanent filtration of 0.9 mm-Al at 75 kV, and a maximum tube voltage of 150 kV. In addition, we used an anthropomorphic thorax phantom (RS-111, Radiology Support Devices, Long Beach, CA, USA) to provide a backscatter medium. This phantom is made of tissue-equivalent materials and represents a thorax of an average male of 175 cm with a weight of 74 kg.

## 3. Results

**Figure 3.**
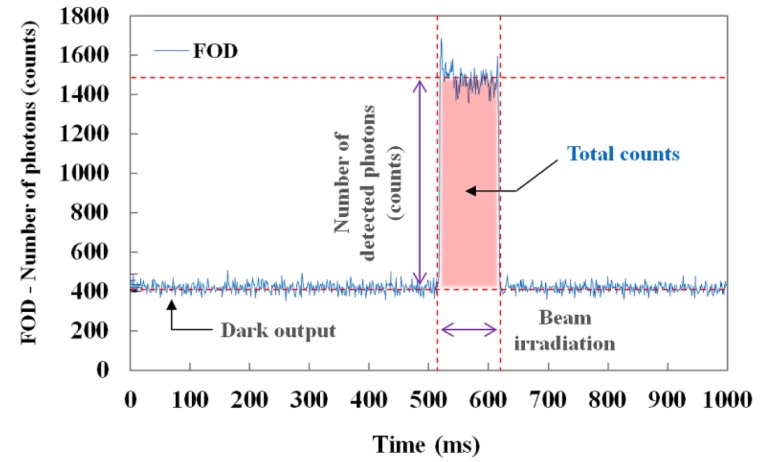
Raw data output from the MPPC module of the FOD system.

Figure 3 illustrates the raw data of real-time output signals from the MPPC module with a threshold level of 0.5 p.e. and a gate time of 1 ms. When the sensing probe was irradiated with an X-ray beam, we measured the number of detected scintillation photons (*i.e.*, counts) in real time and also obtained the total counts of the scintillating light generated from the sensing probe during beam irradiation, as shown in Figure 3.

Through random and repeated experiments to improve accuracy, we measured the scintillating light in order to obtain the dose value, which changed with variations of the exposure parameters, such as the tube potential (kVp), current-time product (mAs), and focus-to-surface distance (FSD) or focus-to-image receptor (*i.e.*, the flat panel detector of the DR system) distance (FID). The scintillation output signals of the FOD were then compared with the dose values simultaneously obtained using the SCD. For this experiment, the values of the tube potential, current-time product (tube current × irradiation time), FSD, and field size were fixed at 100 kVp, 5 mAs (50 mA × 100 ms), 85 cm, and 30 × 30 cm^2^, respectively. During X-ray irradiation, the SCD was placed alongside the sensing probe of the FOD. Error bars were drawn on all data points in the figures showing the experimental results; however, most error bars were within the data points because they were too small to be displayed.

### 3.1. Measurements of the ESD According to the Tube Potential

**Figure 4.**
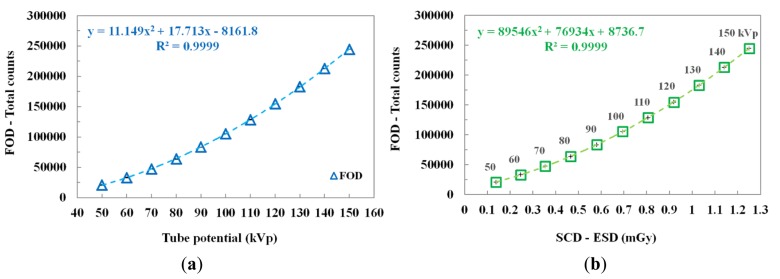
(**a**) Total counts measured by the FOD according to the tube potential. (**b**) Relationship between the total counts of the FOD and the ESDs of the SCD as a function of the tube potential.

The scintillating light and the ESD were measured simultaneously by increasing the tube potential from 50 to 150 kVp in increments of 10 kVp while keeping other exposure parameters constant. As can be seen in Figure 4a, according to the increase of the tube potential, the total counts of the FOD increase according to a quadratic equation because the plastic scintillator has a nonlinear scintillation response to low-energy levels of diagnostic radiation [16,20,21]. Figure 4b plots the relationship between the total counts of the FOD and the ESDs measured using an SCD as a function of the tube potential. The total counts and ESDs increased over the tube potential range as the tube potential increased. The best fit line and the square of the correlation coefficient (R^2^), which refers to the accuracy of matching between the measured datum and the fitting line, are presented in Figure 4.

### 3.2. Measurements of the ESD According to Tube Current

**Figure 5.**
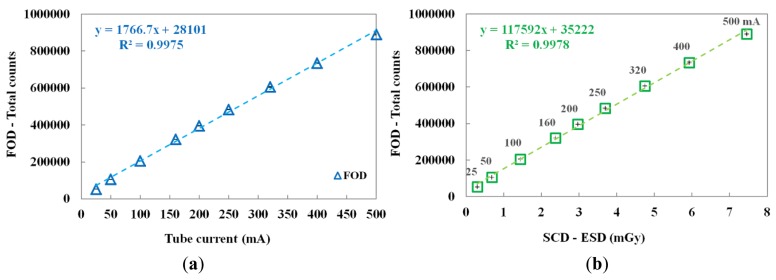
(**a**) Measured total counts using the FOD according to the tube current. (**b**) Relationship between the total counts of the FOD and the ESDs of the SCD at each tube current.

The performance of the FOD was evaluated by increasing the tube current from 25 to 500 mA. In this test, the irradiation time was fixed at 100 ms. Figure 5a,b shows the variation of total counts of the FOD according to the tube current and the relationship between the total counts of the FOD and the ESDs of the SCD at each tube current, respectively.

According to the increase of the tube current, both the total counts of FOD and the ESDs of the SCD linearly increased. Consequently, the proposed miniature FOD has a linear response with respect to the dose rate of the X-ray beam used in diagnostic radiology.

### 3.3. Measurements of the ESD according to Irradiation Time

**Figure 6.**
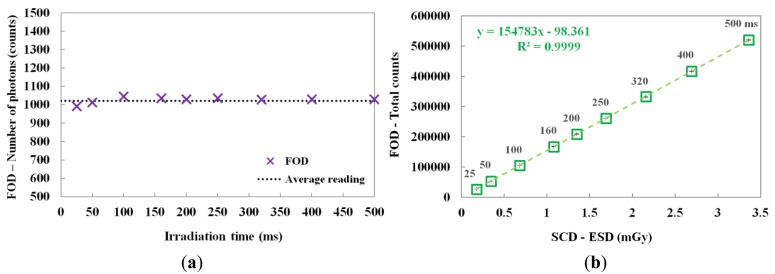
(**a**) Number of scintillation photons measured by the FOD during X-ray irradiation. (**b**) Relationship between the total counts of the FOD and the ESDs of the SCD according to the irradiation time.

By changing the irradiation time, the scintillating light and the ESD were also measured. In this test, the irradiation time of the X-ray beam was varied from 25 to 500 ms while the tube current was fixed at 50 mA. Figure 6a shows the number of scintillation photons measured by the FOD during X-ray irradiation. Although the irradiation time was increased from 25 to 500 ms, the number of detected scintillation photons was almost uniform; however, the total counts of the scintillating light increased linearly with increasing ESD due to the irradiation time, as shown in Figure 6b. The mathematical form of the linear fit line to the coefficient of determination is also presented in Figure 6b and R^2^ was found to be 0.9999.

### 3.4. Performance Evaluation of the FOD with Constant Current-Time Product

**Figure 7.**
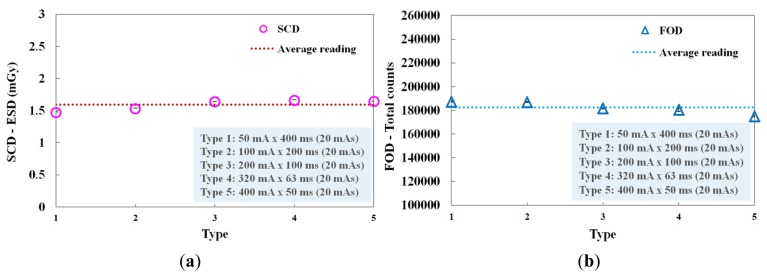
(**a**) ESDs of the SCD and (**b**) total counts of the FOD measured at each current-time product.

We evaluated the performance of the FOD in accordance with the current-time product, whose values were determined by the product of the tube current (mA) and the irradiation time (ms). Figure 7 shows the ESDs of the SCD and the total counts of the FOD measured at each current-time product. In this experiment, although the tube current and the irradiation time were changed, the current-time product was maintained at 20 mAs. Each combination (*i.e.*, type 1∼5) concerning the product of tube current and irradiation time is presented in Figure 7. At type 5, the total counts value of the FOD was slightly lower than the average reading value and it seems that this phenomenon was affected by the relatively small number of scintillation photons detected when the irradiation time was shorter than 50 ms, as can be seen in Figure 6a. However, when each current-time product was fixed, the total counts of the FOD were almost constant due to the decrease of irradiation time with the increase of tube current, as shown in Figure 7b. Therefore, we demonstrated that the total counts of the FOD are only affected by the ESD values even when each exposure parameter is changed independently.

### 3.5. Measurements of the ESD According to Focus-to-Surface Distance

**Figure 8.**
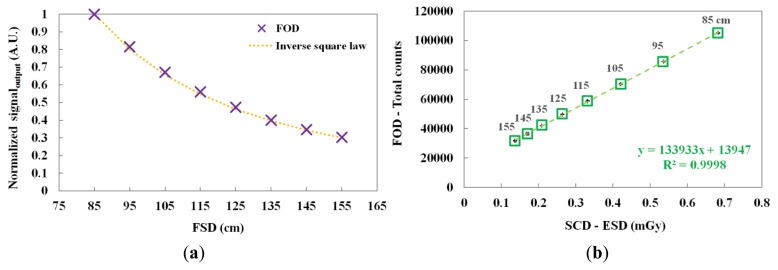
(**a**) Normalized output signals of the FOD at each FSD value. (**b**) Relationship between the total counts of the FOD and the ESDs of the SCD according to the FSD.

The scintillating light and the ESD were measured by increasing the FSD from 85 to 155 cm. Figure 8a,b shows the normalized output signals of the FOD at each FSD and the relationship between the total counts of the FOD and the ESDs of the SCD according to the FSD, respectively. As the FSD increased, the total counts of the FOD and the ESDs of the SCD decreased. In addition, the normalized output signal of the FOD and the theoretical value of the inverse square law were very similar over the distance between the focus of the X-ray tube and the sensing probe placed on top of the thorax phantom.

### 3.6. DR Images of Anthropomorphic Thorax Phantom

**Figure 9.**
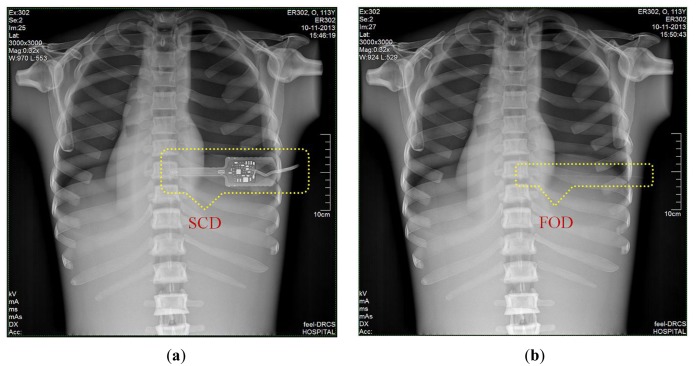
DR images of anthropomorphic thorax phantom with (**a**) a SCD and (**b**) a FOD.

Finally, we obtained DR images from a posteroanterior (PA) chest examination using a flat panel detector of the DR system. In this evaluation, the values of the tube potential, current-time product (tube current x irradiation time), FID, and field size were set at 110 kVp, 10 mAs (320 mA × 32 ms), 180 cm, and 30 × 30 cm^2^, respectively. Figure 9a,b shows two DR images of the anthropomorphic thorax phantom with the finger-shaped dose sensor of the SCD and the sensing probe of the FOD, respectively. To measure real-time ESD and to evaluate the effects of the dosimeter on the image artifacts during PA chest radiography, each distal end of two different dosimeters was placed sequentially at the center of the beam field on the thorax phantom. As can be seen in Figure 9a, the SCD caused image artifacts due to its large size and high-atomic-number materials and, accordingly, the image interfered with the examination of radiation imaging. On the other hand, the FOD makes it possible to measure the ESD with minimization of image artifacts, as shown in Figure 9b, because its size is very small compared to the SCD and it consists of low-atomic materials (*i.e.*, near tissue-equivalent materials) [5]. Particularly, we can expect that the constituent materials of the sensing probe will attenuate X-ray photons in a similar way as the density and atomic compositions of the PSF, POF, and black jacket are quite well matched [22,23].

## 4. Conclusions

In this study, we fabricated a miniature FOD system using a PSF, a POF, and an MPPC module for measuring real-time ESD with minimization of artifacts in the DR image during medical imaging tasks. While varying the exposure parameters of a DR system, we measured the scintillating light related to the ESD using the sensing probe of the FOD, which was placed at the center of the beam field on the anthropomorphic thorax phantom. From the experimental results, the total counts of the proposed FOD system were changed in a manner similar to the ESDs including backscatter simultaneously obtained using a conventional SCD. In particular, we demonstrated that the total counts of the FOD are only affected by the ESD values even though each exposure parameter is changed independently. As functions of each exposure parameter, such as tube potential, tube current, irradiation time, and FSD, the relationships between the total counts of FOD and the ESDs and the best fit lines are presented in Figures 4, 5, 6, and 8 and their R^2^ values are very close to 1. In the range of ESD values less than 1 mGy, the mathematical relation between the total counts of FOD (x) and the ESDs (y) is y = 68939x^2^ + 84559x + 16099, regardless of the exposure parameters. However, this relation has a low accuracy because R^2^ is found to be 0.9794. Therefore, it is necessary to optimize hardware and software of the FOD system for measuring ESD with a high accuracy. Next, DR images were also obtained using a flat panel detector of the DR system to evaluate the effects of the dosimeter on image artifacts during PA chest radiography. The proposed FOD minimally affected the diagnostic information of radiation images while the SCD caused serious image artifacts due to its large size and high-atomic-number materials.

Based on the results of this study, it is anticipated that the proposed miniature FOD system will be a useful dosimeter to measure real-time ESD for diagnostic radiology. Further studies will be carried out to fabricate a multichannel FOD system with PSFs, a POF bundle, and an MPPC array module to simultaneously measure dose distributions in diagnostic applications.
